# Obstetrics

**Published:** 1879-03

**Authors:** 


### OBSTETRICS.

THE UTERO-OVARIAN AMPUTATION AS COMPLEMENTARY TO THE CÆSARIAN OPERATION. H. Debauge. (*Lyon Médical*, Jan. 5, 1878.)

The utero-ovarian amputation in a woman subjected to Cæsarean section, was first practiced at Pavia, May 21, 1875, by Prof.

Porro. In this case it was an operation of necessity, being the only means capable of arresting a hemorrhage which threatened to be speedily fatal. The circumstances surrounding the patient were as unfavorable as possible ; an epidemic of puerperal fever raged in the wards of the maternity, at Pavia ; and yet, the experiment of Porro was crowned with complete success. At the end of six weeks his patient left the hospital, and was able to bear the fatigue of a journey of several hours.

Since this epoch, the example of Porro has had imitators. Today his operation has become the complement of Cæsarean section in Italy, Belgium, Germany and Austria. The accoucheurs of France still hesitate to perform it.

Some months since, one of our young confreres, Dr. Imbert de la Touche, had the happy idea of making the new operation the subject of his inaugural thesis. His remarkable work is before us ; and we think we may render good service in seeking to popularize a method calculated to lessen the mortality in an operation justly regarded as one of the most deplorable resources of surgery.

The Cæsarean operation, as Dr. Imbert remarks, has been performed forty times in Paris, and among these forty operations there has not been a single successful one. More fortunate, the surgeons of *la Charité*, of Lyons, in a series of twenty-five operations made between 1841 and 1878, have had one cure, belonging to Prof. Bouchacourt. A single success opposed to so many deaths, is indeed a sad result ; and there is not another operation in surgery which gives similar statistics.

Among the causes which may be invoked to explain such a frightful mortality, there are three which deserve to be mentioned, because they are annulled by the method of surgeons of Pavia. First, the hemorrhage which follows the operation, a hemorrhage which comes either from the edges of the uterine wound, or from the placental site, and which too often resists all the resources of the operator. Second, the effusion into the peritoneal cavity, of blood, amniotic fluid, and the lochia, an effusion which the utmost precaution cannot prevent. Third, the inflammation which has its origin in the uterus, and from there extends throughout the peritoneum.

To obviate these three causes of death, there is but one resource, the one to which the surgeon of Pavia resorted; the excision of the uterus immediately after the extraction of the child. The necessity of removing with the uterus the entire system of utero-ovarian vessels, and the utility of protecting the patient from the periodic fluxions which the presence of the ovaries entails, led Porro to perform at the same time with the amputation of the uterus that of the ovaries and broad ligaments.

He was encouraged to enter this path by the results following ovariotomy and hysterotomy in affections of the ovary and uterus. The statistics of Péan, of Koeberlé, of Marion Sims, demonstrated to him that the peritoneum can support great injuries better than formerly supposed, and that by making some changes in the process followed in Cæsarean section, it might be possible to make of it something besides a nearly always fatal operation.

The beginning of the operation resembles that of ordinary Cæsarean section, up to the moment when, the uterus being widely open, the surgeon can proceed to the extraction of the child. This done, instead of separating the placenta immediately, the uterus is drawn out through the abominal wound, and kept there by strong traction. The assistants draw the edges of the abdominal wound together, compressing them about the uterine neck. The after-birth is not detached and removed until, by means of these precautions, the penetration of blood into the peritoneal cavity is prevented. The pedicle of the tumor formed by the uterus and its appendages, is clasped by the loop of a linear écraseur, and tightened sufficiently to arrest the flow of blood. All the parts are then removed with the bistoury to within two centimeters of the chain of the écraseur, then the stump thus obtained is traversed by a long needle through its base, which prevents its retraction within the abdominal cavity. The lips of the abdominal wound are united to each other or with the base of the stump by sutures. All the portion of the stump beyond the chain of the écraseur falls at the end of several days, the rest becomes adherent to the abdominal walls, and the cicatrization is complete at the end of a month or six weeks.

Since the 21st of May, 1875, the utero-ovarian operation has been performed fifteen times. An Italian journal, (*Annali uni-*

*versali di Medicina e Chirurgia*, Nov. 1878), gives a statistical table of these fifteen cases; we reproduce it, as it is better calculated to show the advantages of the new method than any theoretic reasoning.

In 1875, utero-ovarian amputaton was performed once only, May 21, at Pavia, by Prof. Porro; the patient, as we have said, got well in six weeks.

In 1876, three operations; two at Vienna by Prof. Spaeth, giving one death and one success; a third, also at Vienna, by Prof. Braun, followed by death.

In 1877, four operations, all followed by death; one at Milan by Prof. Chiara; a second at Fribourg by Prof. Hegar; a third at Bergamo, by Dr. Previtali; the fourth at Berne, by Prof. Muller.

In 1878, seven operations; at Milan, by Prof. Chiara, two cases, one death and one cure; at Turin, Prof. Tibino, one death; at Liege, Prof. Wasseige, one cure and one death; at Prague, Prof. Breisky, one death; at Brescia, Dr. Peroglio, one cure.

Seven cures and eight deaths; a result not sufficient to remove the dread of Cæsarean section, but when compared with the old plan we cannot help recognizing that the process of Porro constitutes an important progress, one deserving its adoption in the practice of our hospitals.

